# Formulation of Neem oil-loaded solid lipid nanoparticles and evaluation of its anti-*Toxoplasma* activity

**DOI:** 10.1186/s12906-022-03607-z

**Published:** 2022-05-04

**Authors:** Sara Nemati, Hanieh Mohammad Rahimi, Zahra Hesari, Meysam Sharifdini, Nooshin Jalilzadeh Aghdam, Hamed Mirjalali, Mohammad Reza Zali

**Affiliations:** 1 Foodborne and Waterborne Diseases Research Center, Research Institute for Gastroenterology and Liver Diseases, Shahid Beheshti University of Medical Sciences, Tehran, Iran; 2 Department of Pharmaceutics, School of Pharmacy, Guilan University of Medical Sciences, Rasht, Iran; 3 Department of Medical Parasitology and Mycology, School of Medicine, Guilan University of Medical Sciences, Rasht, Iran; 4 Madani Hospital, Alborz University of Medical Sciences, Karaj, Iran; 5 Gastroenterology and Liver Diseases Research Center, Research Institute for Gastroenterology and Liver Diseases, Shahid Beheshti University of Medical Sciences, Tehran, Iran

**Keywords:** Acute toxoplasmosis, Alternative medicine, Neem oil, Solid lipid nanoparticles, *Toxoplasma gondii*

## Abstract

**Background:**

Toxoplasmosis is caused by an intracellular zoonotic protozoan, *Toxoplasma gondii*, which could be lethal in immunocompromised patients. This study aimed to synthesize Neem oil-loaded solid lipid nanoparticles (NeO-SLNs) and to evaluate the anti-*Toxoplasma* activity of this component.

**Methods:**

The NeO-SLNs were constructed using double emulsification method, and their shape and size distribution were evaluated using transmission electron microscope (TEM) and dynamic light scattering (DLS), respectively. An MTT assay was employed to evaluate the cell toxicity of the component. The anti-*Toxoplasma* activity of NeO-SLNs was investigated using vital (trypan-blue) staining. Anti-intracellular *Toxoplasma* activity of NeO-SLNs was evaluated in *T. gondii-*infected Vero cells.

**Results:**

The TEM analysis represented round shape NeO-SLNs with clear and stable margins. DLS analysis showed a mean particle size 337.6 nm for SLNs, and most of nanoparticles were in range 30 to 120 nm. The cell toxicity of NeO-SLNs was directly correlated with the concentration of the component (*P*-value = 0.0013). The concentration of NeO-SLNs, which was toxic for at least 50% of alive *T. gondii* (cytotoxic concentration (CC_50_)), was > 10 mg/mL. The ability of NeO-SLNs to kill *Toxoplasma* was concentration-dependent (*P*-value < 0.0001), and all concentrations killed at least 70% of alive tachyzoites. Furthermore, the viability of *T. gondii-* infected Vero cells was inversely correlated with NeO-SLNs concentrations (*P*-value = 0.0317), and in the concentration 100 μg/mL at least 75% of *T. gondii-* infected Vero cells remained alive.

**Conclusions:**

Overall, our findings demonstrated that the NeO-SLNs was able to kill *T. gondii* tachyzoites in concentration 100 μg/mL with a cell toxicity lower than 20%. Such results suggest that employing SLNs as carrier for NeO can effectively kill *T. gondii* tachyzoites with acceptable cell toxicity. Our findings also showed that SLNs capsulation of the NeO can lead to prolonged release of the extract, suggesting that NeO-SLNs could be also employed to clear cyst stages, which should be further investigated in animal models.

## Background

*Toxoplasma gondii* is an intracellular protozoan parasite with worldwide distribution (seropositivity rate up to one third of world’s population), which infects almost all vertebrates [1, 2]. The transmission routs of this parasite are mainly ingestion of either oocysts from contaminated water, food, vegetables, and fruits, or tissue cysts in undercooked meat. Toxoplasmosis is a common opportunistic infection in immunocompromised patients such as HIV/AIDS patients, which may cause encephalitis [3, 4], chorioretinitis [5], and even death.

The first-line recommended treatment for toxoplasmosis is a combination of pyrimethamine and sulfadiazine with Leucovorin [6]. However, this combination is not effective enough in cases who suffer from encephalitis, chorioretinitis, and congenital toxoplasmosis [6–8]. On the other hand, because of adverse effects, the prescription of these drugs has been limited [9]. Therefore, less toxic drugs are being developed as an alternative treatment plan in immunocompromised patients with toxoplasmosis.

Over last decade, a broad-spectrum of herbal drugs and plant extracts have been used as alternative therapy [10–12] to reduce the side effects of chemical medicines. The Neem oil (NeO) is extracted from *Azadirachta indica* and was considered to be a potential source for developing new drugs to overcome limitations of conventional chemical agents [13]. The NeO is a natural pesticide with promising outcomes on skin diseases [14–16], which has also showed anti-hemorrhoid, anti-protozoal, and anti-bacterial effects [15, 17]. The NeO and its components have been reported to be effective on broad-spectrum of bacteria [18–21], and its effects on herpes virus [22] and the intracellular *Toxoplasma* [23] also suggested promising results.

During recent years, nano-formulations of the NeO, mostly as pesticides, have been fabricated and developed to improve the functionality and decrease toxicity of this natural component [14, 24, 25]. Nano carriers such as solid lipid nanoparticles (SLN) have been suggested to promisingly increase the penetration capability and the effectiveness of drugs [26, 27]. Therefore, the present study aimed to synthesize Neem oil-loaded solid lipid nanoparticles (NeO-SLNs) and to evaluate the cell toxicity and anti-*Toxoplasma* activity of this component.

## Methods

### Materials

Soya lecithin (DUKSAN reagents, South Korea), cholesterol (Sigma, Germany), PVA (Merck, Germany), and Tween 80 and dichloromethane (both with analytical grade; Merck, Germany) were purchased. The Neem oil was purchased from local market in Rasht, Guilan province, Iran.

### Methods

#### Preparation of Neem oil loaded solid lipid nanoparticles

In this study, cholesterol was used as lipid, and lecithin and Tween 80 were as surfactants. To prepare NeO-SLNs, double emulsification method (w/o/w type) was employed [28]. Briefly, 1 mL of the NeO was dissolved into 10 mL aqueous mixture of methanol (75% v/v). Then, 100 mg of lecithin and 100 mg of cholesterol were dissolved in dichloromethane. The NeO solution was slowly added to the lipid mixture and homogenized for 15 min at 15000 rpm in an ultra-probe sonicator (The ultrasonic processor UP400; Hielscher, Germany) to produce white cloudy primary emulsion. The resultant primary emulsion was mixed with 4% w/v of PVA solution and homogenized for an additional 10 min at 15000 rpm. The resultant w/o/w was subjected to a rota evaporator at 45 °C to completely evaporate the organic solvent. Next, the stable emulsion was freeze dried (Emulsion was freezed at − 20 °C and were subjected to under air vacuum for ice sublimation overnight) using a freeze dryer (ZIRBUS, VaCo 5-II-D, Germany) to get dried powder of NeO-SLNs.

#### Electron microscopy

The morphology of the nanoparticles was investigated using a transmission electron microscope (TEM) (EM10C-100 KV; Carl Zeiss, Germany) operated at 80 kV. Drops of the nanoparticle suspensions, contrasted with uranyl acetate, were placed on 200–300 mesh grids coated with Formar (a low absorption resin). The grids were analyzed after being allowed to dry by evaporation.

#### Particle size, zeta potential and poly dispersity index (PDI) measurement

A Malvern particle size analyzer (Zetasizer 1,033,439, Malvern Instrument, UK) was employed to characterize the size of nanoparticles. Briefly, 15 μL of SLNs were suspended in 1 mL double distilled water (DDW) [29]. The experiment was performed at 25 °C with a count rate of 206.3 kcps and measurement position of 4.65 mm. The average particle size was calculated using Zetasizer Ver. 6.01 software. The zeta potential measurement of nanoparticles was determined by Zetasizer 1,033,439 (Malvern Instrument, UK), and zeta potential value was expressed in mV. The polydispersity index value was determined to evaluate the monodisperse or polydisperse nature of the prepared nanoparticles. High polydispersity index values indicate a high level of non-uniformity.

#### Entrapment efficiency

100 mg of SLNs was dissolved in 20 mL of dichloromethane and the solution was centrifuged at 12000 rpm. The supernatant was collected and passed through a membrane filter. The quantity of oil in the solution was measured by UV spectroscopy (PerkinElmer, USA) at 205 nm [30]. Entrapment efficacy was calculated using the following formula:

$$\mathrm{Entrapment}\;\mathrm{efficiency}\;(\%)=\mathrm{Quantity}\;\mathrm{of}\;\mathrm{drug}\;\mathrm{in}\;\mathrm{nanoparticle}/\mathrm{Mass}\;\mathrm{of}\;\mathrm{drug}\;\mathrm{in}\;\mathrm{the}\;\mathrm{formulation}\times100$$

#### Fourier transform infrared (FTIR) analysis

The pure NeO and SLNs were subjected to FTIR spectrometry (Version 10.5.3; PerkinElmer ES, USA) to examine the probable incompatibilities between the NeO and incorporated excipients. FTIR spectra for the NeO and NeO-SLNs were collected at a resolution of 4 cm^− 1^ and given as the ratio of 21 single beam scans to the same number of background scans in pure KBr.

#### In-vitro release and release kinetic study

In-vitro release of the NeO-SLNs was investigated using dialysis bag method. Briefly, a 14 kDa dialysis tubing cellulose membrane (CM) was soaked in distilled water for 24 h. Then, 1 g of SLNs was packed in dialysis tube and placed in 100 mL phosphate buffered saline (PBS; 0.1 M; pH = 6.8), as receptor medium, in room temperature. The medium was stirred at 100 rpm during the release test and samples were withdrawn at certain time intervals of 0, 1, 6, 24, 48 and 72 h. The release study was performed for 72 h to be able to analyze the release rate and kinetic in a relatively longer period. The NeO contents were then analyzed by UV spectrophotometry in 205 nm. To investigate the release kinetics of the NeO-SLNs, the in vitro release data was analyzed using various mathematical kinetic models including Zero and First orders, Higuchi, and Korsmeyer-Peppas using freeze dried SLNs, as mentioned elsewhere [31, 32].

### Biological experiments

#### Cell culture

In order to in vitro assay, the kidney fibroblast from the African green monkey (Vero; ATCC: CCL-81) cells, which was kindly gifted by the Institute Pasteur of Iran, were cultivated in Dulbecco’s Modified Eagle Medium (DMEM; Biosera, Arya Tous, Iran), supplemented with 10% heat-inactivated FBS (Gibco, Thermo Fisher Scientifc, MA, USA) and 1% Antibiotic-Antimycotic (100X) (1% penicillin/streptomycin; Gibco, Thermo Fisher Scientifc, MA, USA), and were maintained at 37 °C in 5% CO_2_.

#### Cell toxicity assay for the Vero cell

To assay the cell toxicity effects of NeO-SLNs, 1 × 10^5^ Vero cells were seeded in 96-well plates in DMEM medium without antibiotics and incubated at 37 °C in 5% CO_2_ for 24 h. Then, serial dilutions (log^− 10^ from 100 mg/mL to 100 μg /mL) of the NeO-SLNs were added to each well and incubated at 37 °C in 5% CO_2_ for 2 days. The viability rate of the Vero cell against serial dilutions of NeO-SLNs was evaluated using MTT (3-(4,5-dimethylthiazol-2-yl)-2, 5-diphenyltetrazolium bromide) assay. Wells of the Vero cells without treatment and a treated with clindamycin (150 mg/mL, Zahravi, Iran) were assigned as negative and positive controls, respectively.

After 24 h, the supernatant was removed and 15% (v/v) of the MTT solution (5 mg/mL) was directly added to the wells, and the plate was incubated for additional 4 h at 37 °C in 5% CO_2_ before stopping the experiments by dimethyl sulfoxide (DMSO; Me_2_SO; 150 μL/well, Merck, Germany). To evaluate the results, plate was read using an enzyme-linked immunosorbent assay (ELISA) microplate reader (LX800; Biotec, Winooski, VA, USA) at wavelength 570. All experiences were done in duplicate, and the toxicity effects were calculated using the formula, which was mentioned elsewhere [33–36]. The 50% Inhibitory concentration (IC_50_) was calculated for concentration that killed at least 50% of *T. gondii*. In addition, the concentration, which was toxic for at least 50% of host cells, was considered as 50% cytotoxic concentration (CC_50_).

#### *T. Gondii* strain

Tachyzoites of *T. gondii* (RH strain), which were kindly gifted by Dr. Seyed Tabaei, were washed with sterile PBS (pH: 7.4), counted by hemocytometer slide, and incubated with the Vero cells (1.5 × 10^6^ tachyzoites with ∼10^5^ of the Vero cells; [multiplicity of infection (MOI) = 10]). The cells were cultivated in DMEM supplemented with 10% FBS and 1% penicillin/streptomycin, for mass cultivation [33, 34].

#### Anti-*Toxoplasma* activity of the NeO-SLNs

To evaluate the anti-*Toxoplasma* activity of the NeO-SLNs, six serial dilutions of NeO-SLNs (log^− 10^ from 1 mg/mL to 100 μg/mL) and Clindamycin (150 mg/mL, Zahravi, Iran) (as positive control) were added to 1 × 10^6^ tachyzoites. A well of *T. gondii* without any treatment was negative control. After 2 h, 10 μL of *T. gondii* from each well of different concentrations of the NeO-SLNs were stained by vital staining (trypan blue), and the number of alive cells was calculated using hemocytometer slide [36, 37]. All experiments were performed in duplicate. To calculate the toxicity value of the NeO-SLNs, the published formula was employed [33].

#### Ratio

To calculate the probable best concentration of the NeO-SLNs with the highest anti-*Toxoplasma* activity and lowest cell toxicity, the ratio was calculated as described previously [33, 36].

### Statistical analysis

GraphPad Prism software (version 8.3.0.538) was employed to statistically analyze the results. One-sample *t-*test was employed to evaluate the statistical correlations among samples.

### Intracellular anti-*Toxoplasma* activity

To evaluate the effects of the NeO-SLNs on the intracellular *Toxoplamsa*, we employed the protocol described by Khosravi et al. [33]. Briefly, 1 × 10^5^ of the Vero cells were seeded in a 96-well cell culture plate containing DMEM supplemented with 10% FBS without antibiotics. The confluent Vero cells were incubated with 1 × 10^5^ of *T. gondii* tachyzoites RH strain (MOI = 1) at 37 °C and 5% CO_2_ for 24 h. Afterwards, different concentrations (log^− 10^ from 1 mg/mL to 100 μg /mL) of the NeO-SLNs were added to each well, and the plate was additionally incubated at 37 °C and 5% CO_2_ for 24 h. Finally, the supernatant was removed, and wells were washed twice by sterile PBS to remove cell debris, dead cells, and extracellular tachyzoites. A well of Me_2_SO was used as a control. Then, the wells were checked to confirm the maximum cellular invasion by the parasites. Finally, the culture supernatant was removed and the MTT assay was performed, and the absorbance of the plates at 570 nm was read with ELISA reader (LX800; Biotec, Winooski, VA, USA) [33].

## Results

### Fabrication of the NeO-SLNs, electron microscopy, and particle size analysis

**Fig. 1:**
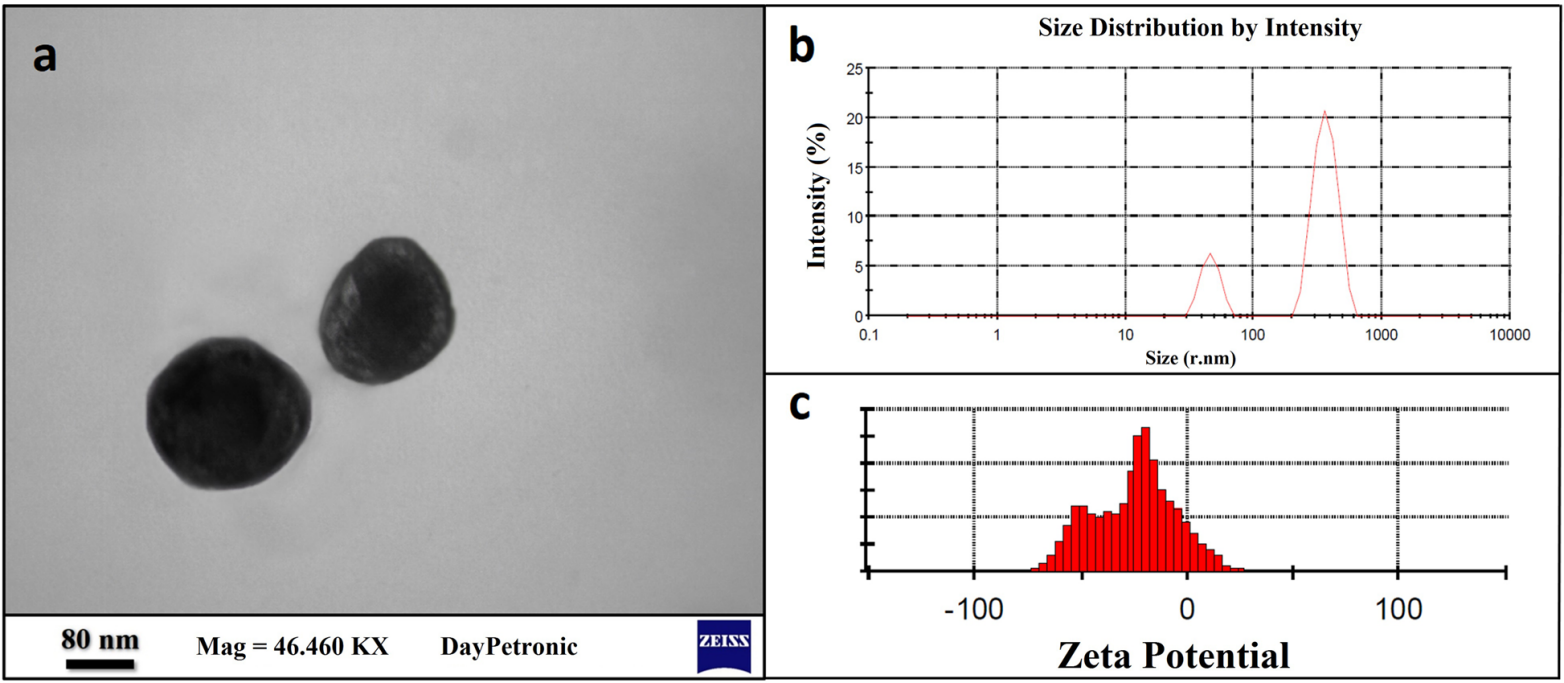
**a** TEM images of NeO-SLNs with magnification of 46.460 KX shows two single nanoparticles with higher magnification. **b** DLS analysis reported two main peaks for SLN particle size in nano range: 365.9 nm and 46.16 nm. **c** Zeta potential and PDI were − 26.5 mV and 0.77, respectively, confirming a polydispersed and stable formulation

The NeO-SLNs were successfully synthesized. TEM analysis showed that the NeO-SLNs were round shape with clear and stable margins (Fig. 1a). DLS analysis reported two main peaks for SLN particle size in nano range: 365.9 nm and 46.16 nm with the calculated mean particle size of 337.6 nm (Fig. 1b). Results demonstrated the zeta potential and PDI were − 26.5 mV and 0.77 respectively (Fig. 1c), confirming a polydispersed and stable formulation.

### Entrapment efficiency

Based on mentioned calculations, prepared NeO-SLNs showed the entrapment efficiency equal to 71.61 ± 2.36%. Relatively high %EE can be attributed to the lipophilic nature of drug, as it had higher affinity for the lipid matrix.

### FTIR- spectra (compatibility with excipients)

**Fig. 2:**
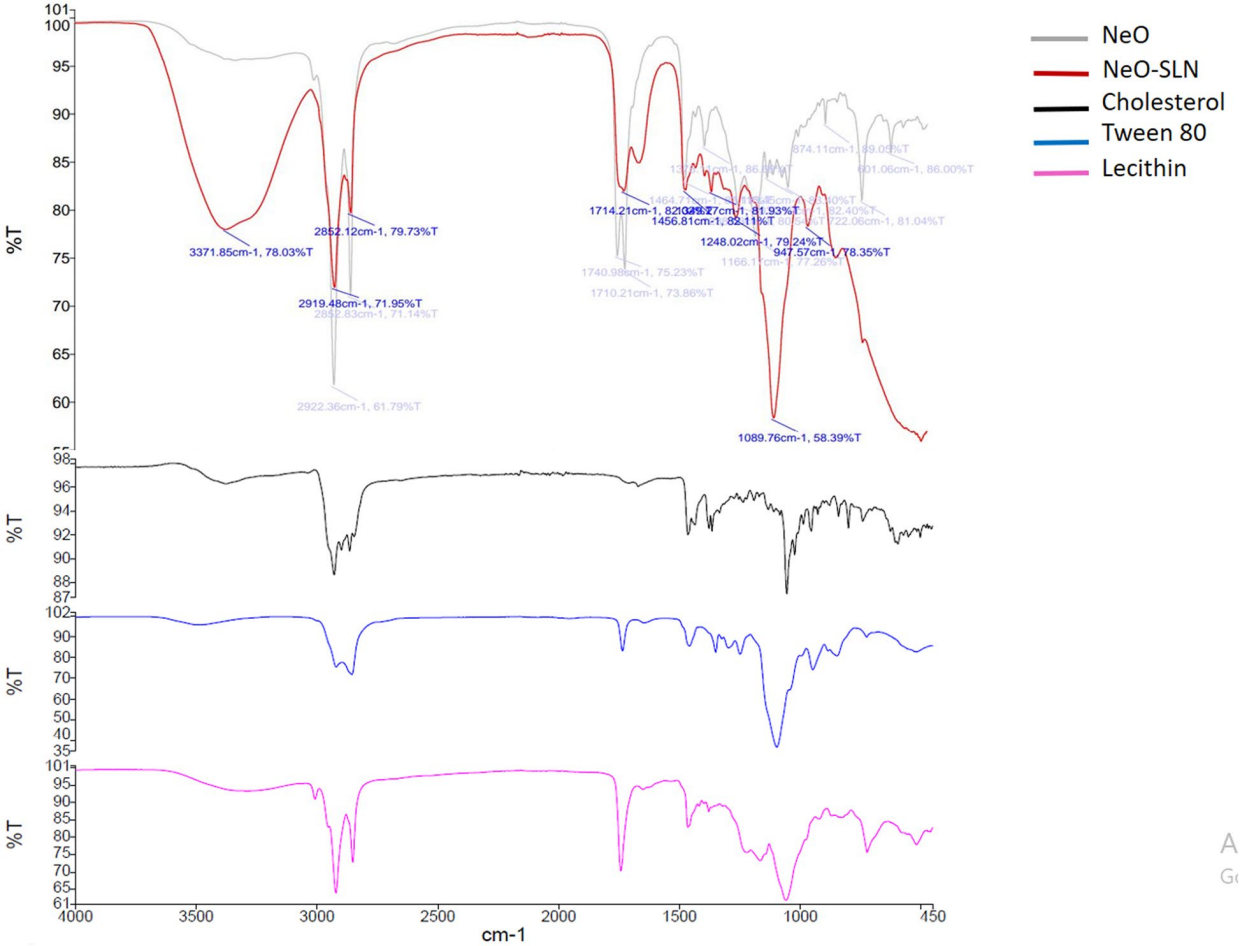
The main structure of the peaks of the NeO spectrum including C-H aliphatic doublet peak on 2919 and 2852 and C=O doublet peak on 1712 and C-H bending peak on 1456

The NeO and NeO-SLNs were subjected to the FTIR. The main structure of the peaks of the NeO spectrum was also observed in the NeO-SLNs spectrum including C-H aliphatic doublet peak on 2919 and 2852 and C=O doublet peak on 1712 and C-H bending peak on 1456 (Fig. 2), which all matched with existing NeO spectra in previous studies [38, 39]. Indeed, no specific interaction was observed between the NeO and the lipids used in the formulations. In addition, the FTIR spectrum of SLN excipients (cholesterol, lecithin and tween 80) is also demonstrated.

### In vitro release and release kinetic studies

**Fig. 3:**
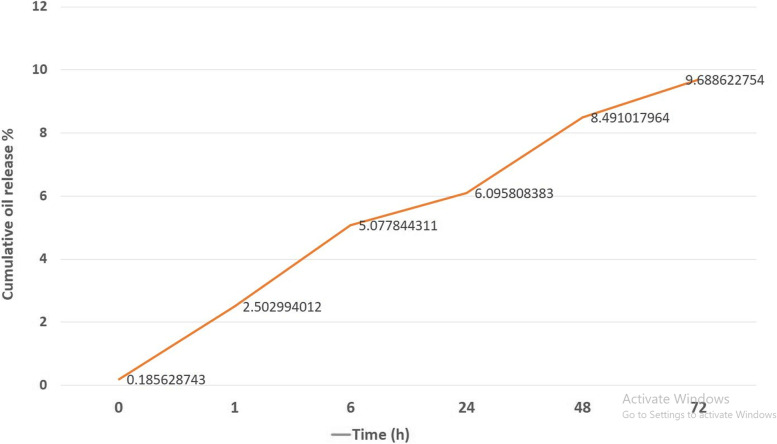
Drug release of the formulation. Maximum cumulative was 9.67% in 72 h, which reveals a sustain controlled release of the NeO for long term with no initial burst release

The in vitro release studies of NeO-SLNs was evaluated by reverse dialysis bag. The amounts of drug release of the formulation were showed in Fig. 3. Maximum cumulative release reached to amount of 9.67% in 72 h, which reveals a sustain controlled release of the NeO for long term with no initial burst release.

The values of zero order, first order, Higuchi, and Korsemeyer-Peppas models, were obtained and listed (Table 1). The highest regression coefficient (R^2^) was considered as the best fitted kinetic model for the formulation. Drug release kinetic in the SLNs showed the highest regression coefficient with Korsemeyer-Peppas model with *n* = 0.461 (Table 1). The release mechanism of the NeO from the SLNs would be based on anomalous (non-Fickian) diffusion, which suggests coupling of diffusion and erosion mechanisms (anomalous diffusion), and controlling of the drug release by at least two processes.

**Table 1:** Release kinetic parameters for the NeO-SLN based on different mathematical models

Formulation	Zero order		First order		Higuchi		Korsemeyer-peppas	
	K_0_	R^2^	K_1_	R^2^	K_H_	R^2^	n	R^2^
NeO-SLN	29.314	0.9168	−0.4095	0.8084	29.314	0.9168	0.461	0.9237

### Cell toxicity of the NeO-SLNs

**Fig. 4:**
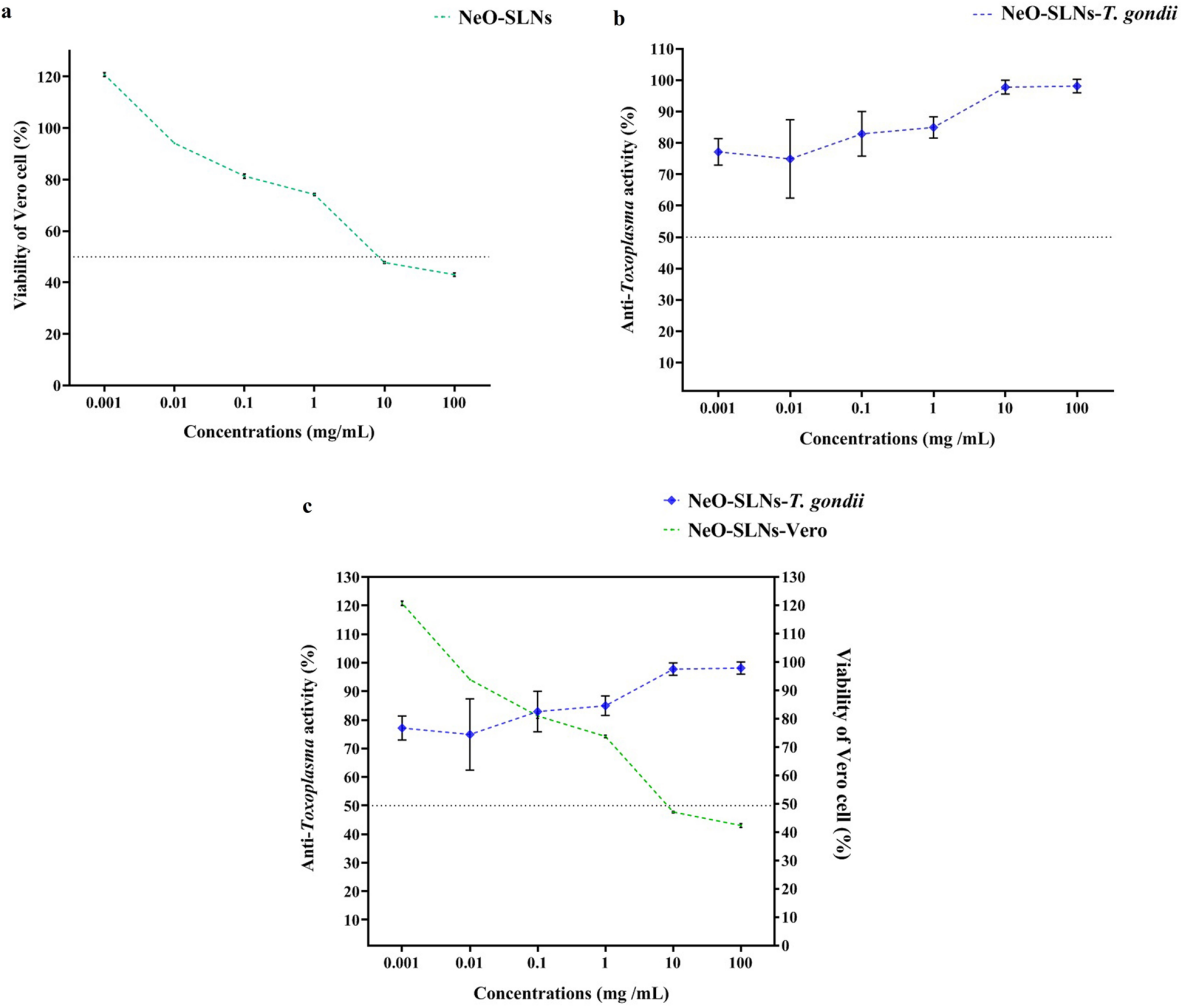
**a** Cell toxicity assay of NeO-SLN on Vero cell line. The statistical significant decrease was seen in the toxicity of NeO-SLNs regarding the concentration (*P*-value = 0.0013). As well, the analysis indicated CC_50_ > 10 mg/mL. **b** Anti-*Toxoplasma* activity of NeO-SLNs according to different concentrations. One-sample *t-*test indicated a statistically significant anti-*Toxoplasma* activity of NeO-SLNs (*P*-value < 0.0001) with IC_50_ > 1 μg/mL. **c** Ratio analysis of NeO-SLNs suggests that the concentration 100 μg/mL with a value close to 1 has high anti-*Toxoplasma* activity and low Vero cell toxicity

The cell toxicity assay indicated that The CC_50_ value for the NeO-SLNs was at the concentrations > 10 mg/mL. Accordingly, at the concentration of 10 mg/mL and 100 mg/mL of the NeO-SLNs 47.770 + 0.325% (95% CI: 47.540 to 48.00%) and 43.060 + 0.693% (95% CI: 42.570 to 43.550%) of the Vero cells remained viable, respectively. Nevertheless, at the lower concentration of the NeO-SLNs (1 μg/mL), viable Vero cell were 120.785 + 0.757% (95% CI: 120.250 to 121.320%). The statistical analysis showed a reduced toxicity of the NeO-SLNs regarding the concentrations (*P*-value = 0.0013) (Fig. 4a; Table 2).

**Table 2:** Anti-intracellular *Toxoplasma* activity of different concentrations of NeO-SLN

Concentrations mg/mL	Anti-***Toxoplasma*** activity		***P***-value	Cell viability		***P***-value	Ratio
	Mean **+** SD (%)	95% CI		Mean **+** SD (%)	95% CI		
100	98.14 + 2.148	96.280 to 100	< 0.0001	43.060 + 0.693	42.570 to 43.550	0.0013	2.280
10	97.76 + 2.191	94.79 to 100		47.770 + 0.325	47.540 to 48.000		2.046
1	84.94 + 3.401	81.41 to 89.59		74.225 + 0.431	73.920 to 74.530		1.149
0.1	82.89 + 7.103	74.72 to 91.07		81.385 + 0.870	80.770 to 82.000		1.026
0.01	74.90 + 12.49	57.62 to 87.36		94.110 + 0.000	94.110 to 94.110		0.795
0.001	77.13 + 4.22	71 to 80.67		120.785 + 0.757	120.250 to 121.320		0.638

*Note*: One-sample t-test was employed to evaluate the statistical correlations among samples

### Anti-*Toxoplasma* activity of the NeO-SLNs

Toxic values of the NeO-SLNs changed the morphology of *T. gondii* tachyzoites in vital staining. The results of anti-*Toxoplasma* activity showed that the NeO-SLNs was able to kill at least 70% of *T. gondii* tachyzoites at all concentrations. In addition, one-sample *t-*test indicated a statistically significant concentration-dependent anti-*Toxoplasma* activity of the NeO-SLNs (*P*-value < 0.0001). The lowest and highest concentrations killed 77.13 + 4.22 (95% CI: 71 to 80.67) and 98.14 + 2.148 (95% CI: 96.280 to 100), respectively with an IC_50_ > 1 μg/mL (Fig. 4b; Table 2).

### Anti-intracellular *Toxoplasma* activity of the NeO-SLNs

**Fig. 5:**
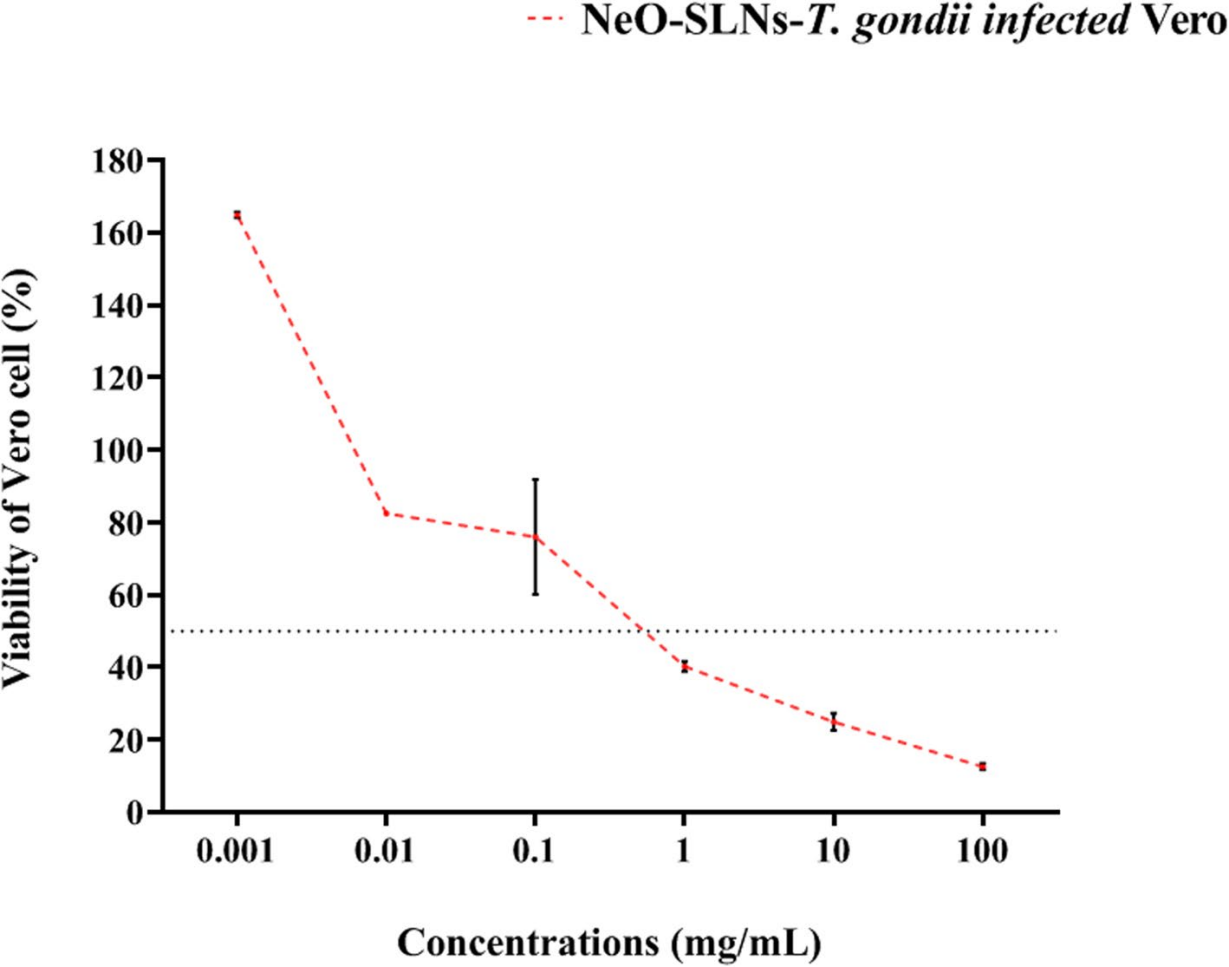
Anti-*Toxoplasma* activity of different concentrations of NeO-SLN*.* The results showed statistically significant anti-intracellular *Toxoplasma* activity of NeO-SLN (*P*-value = 0.0317), while more than 75% of *Toxoplasma*-infected Vero cells remained viable in concentrations ≤100 μg/mL

The results of anti-intracellular *Toxoplasma* activity of the NeO-SLNs showed an increased cell viability regarding log ^− 10^ of the NeO-SLNs (*P*-value = 0.0317). In another word, more than 75% of *Toxoplasma*-infected Vero cells remained viable in concentrations ≤100 μg/mL (Fig. 5; Table 3). These results showed the uptake of NeO-SLNs by Vero cells. The viability of Vero cell at concentrations ≤100 μg/mL showed that Vero cells are able to uptake NeO-SLNs even in sub-milligram concentration values of the component.

**Table 3:** Anti-*Toxoplasma* activity of NeO-SLNs

Concentrations mg/mL	Anti-intracellular ***Toxoplasma*** activity		***P-***value
	Mean **+** SD (%)	95% CI	
100	12.520 **+** 0.877	11.900 to 13.140	0.0317
10	24.925 **+** 2.355	23.260 to 26.590	
1	40.260 **+** 1.372	39.290 to 41.230	
0.1	75.990 **+** 15.910	64.740 to 87.240	
0.01	82.465 **+** 0.078	82.410 to 82.520	
0.001	164.830 **+** 0.820	164.250 to 164.410	

*Note*: One-sample t-test was employed to evaluate the statistical correlations among samples

### Ratio analysis

The comparison of the efficacy to safety of the NeO-SLNs (here called ratio) suggested that the concentration 100 μg/mL (with ratio 1.026) had the highest anti-*Toxoplasma* activity 82.89 + 7.103 (95% CI: 74.72 to 91.07) and lowest cell toxicity against the Vero cell 81.385 + 0.870 (95% CI: 80.770 to 82.000) (Fig. 4c; Table 2).

## Discussion

During recent years, due to the non-availability of safe and effective drugs, side effects of the current drugs [40], the emerging drug-resistant strains [41, 42], and the contribution of the ethno-pharmacological knowledge for alternative medicine, there is a necessity for development of new drugs.

Although scientific evidence indicates that the safety and efficacy of herbal remedies are limited, herbal drugs are being consumed due to their low undesirable side effects and toxicity, as well as their low price [42, 43]. Furthermore, in the case of low effectiveness of conventional drugs, there is an increased usage of traditional herbal remedies [41, 44, 45]. Over last decade, many studies have investigated the effects of medicinal plants on protozoa [10, 44, 46–48] and helminths [49–52].

*T. gondii* is an opportunistic and life-threatening parasite, particularly in AIDS patients, cancer patients, and organ transplant recipients [3, 7]; therefore, treatment of human toxoplasmosis seems to be an urgent need for susceptible groups. Several studies have evaluated the effects of herbal medicines on *T. gondii* [10, 36, 53–56]. *A. indica* (Neem) belongs to Mahogany family and has been used as herbal medicine, particularly in Africa and south of Asia [57]. The Neem extracts have exhibited immunomodulatory, anti-hyperglycemic, anti-carcinogenic, anti-parasitic, antiviral, insecticidal, and antioxidant properties [16, 17, 19, 58–60]. Blum et al. [19] evaluated the anti-*Helicobacter pylori* effects of the NeO and suggested that this oil has promising bactericidal effects and would be considered for further treatment strategies against this bacterium. However, there is a little data about the effects of the Neem extract on *T. gondii*. In this regard, Melo et al. [23] evaluated the effects of the aqueous extract of Neem on intracellular development of *T. gondii* in Vero cell, and observed a dramatically decrease in the number of the parasite in the parasitophorous vacuole without significant changes in the host cells. Nevertheless, due to some impediments, the wide biological usage of the Neem extracts has been limited. Actually, unspecific standard dosage, low stability, unknown side effects, low bioavailability, and low available scientific evidence seem to be the main concerns for the commercial development of the herbal medicine [61]. For example, Mohammad Rahimi et al. [36] showed that although the aqueous extract of *Mentha pulegium* L. and *Rubus idaeus* L. presented promising anti-*Toxoplasma* effects, cell toxicity of the extracts, particularly, *R. idaeus* L. limited employing of *R. idaeus* L. for herbal therapy. The nanoformulation of the herbal extracts can not only increase the bioavailability, effectiveness, and target delivery, but also decrease the aforementioned limitations of the herbal extracts [9, 62]. In the current study, we employed the SLNs to capsulate the NeO and increase its target delivery and efficiency. The SLNs are a group of nano-particles that are used to control the release and target delivery of drugs [63]. In addition, this type of encapsulation increases the efficiency of natural products [63, 64]. The SLNs, as a carrier, are reported to effectively increase the bioavailability of commercial drugs. Ud Din et al., [29] demonstrated that SLNs formulation of ezetimibe increased the bioavailability of this drug compared to marketed product, while its stability remained without significant changes for three months.

The previous study in our laboratory suggested the promising effects of SLN capsulation. In this regard, Khosravi et al. [33] suggested that the SLN capsulation of paromomycin decreased the cell toxicity and increased anti-*Toxoplasma* activity of the drug. Similar to our protocol, Vijayan et al., [28] synthesized NeO-SLNs with a high entrapment efficacy (82.1%) and investigated the anti-Acne microbes activity of NeO-SLNs that due to the promising results, they continued prolonged treatment of Acne. In addition, the results of a study by Kim et al., [65] who formulated SLNs to carry *Houttuynia cordata* for oral delivery, showed a low cell toxicity of SLNs loaded *H. cordata* on caco-2 cell line, while its cumulative release continued to 50 h. The more effective delivery of SLNs loaded herbal extracts was also suggested by Vijayanand et al. [66], who proposed encapsulation of *Hibiscus rosa sinensis* extract by SLNs improve efficacy of the extract compared to crude extract, even at low dosage and in vivo model. In the line of these studies, our findings showed suitable anti-*T. gondii* activity besides low cell toxicity in 100 μg/mL concentration of the NeO-SLNs. In addition, the NeO-SLNs promisingly limited the intracellular development of *Toxoplasma* in Vero cells in 100 μg/mL concentration. Moreover, high entrapment and satisficing cumulative release of NeO-SLNs in our study suggested that encapsulation of the NeO by SLNs could be effective as an alternative therapy for not only *T. gondii* tachyzoites, which are released in acute phase of toxoplasmosis, but also for cysts, which are formed in chronic stage. However, further studies are needed to evaluate the effects of NeO-SLNs in animal models and non-lethal (cyst-forming) strains.

## Conclusions

Our findings demonstrated that the NeO-SLN was able to kill *T. gondii* tachyzoites in concentration 100 μg/mL. Interestingly, the best concentration with lowest cell toxicity and highest anti-*Toxoplasma* activity was same with the IC_50_ value for anti-intracellular *Toxoplasma* activity. Our findings also showed that SLNs capsulation of the NeO can lead to prolonged release of the extract. Overall, our results suggest that nanoformulation of natural products can increase efficiency and decrease toxicity of the herbal component such as the NeO to add them in treatment strategies as alternative medicine. As well, prolonged effective release of the NeO suggests that NeO-SLNs could be employed to clear cyst stages, which should be further investigated in animal models.

## Data Availability

All datasets generated for this study are included in the article/supplementary files.

## Abbreviations

CC_50_: 50% cytotoxic concentration
DLS: Dynamic light scattering
DMEM: Dulbecco’s Modified Eagle Medium
NeO: Neem oil
SLN: Solid lipid nanoparticle
NeO-SLNs: Neem oil-loaded solid lipid nanoparticles
MOI: Multiplicity of infection
PVA: Polyvinyl alcohol
TEM: Transmission electron microscope
